# Frederic Percival Mackie

**Published:** 1944

**Authors:** 


### FREDERIC PERCIVAL MACKIE, C.S.I., O.B.E., M.D., M.Sc., D.P.H. (Bristol), F.R.C.S.

An old Bristol student of very great distinction has recently passed away in Colonel F. P. Mackie. He was son of a late rector of Filton. Educated at Dean Close School, Cheltenham, Mackie entered the Bristol Medical School and University College in 1892, qualifying M.R.C.S., L.R.C.P., in 1897. He took his F.R.C.S. England in 1902, and after the University of Bristol was established he obtained the degrees of M.Sc. and M.D., as well as the D.P.H. in that University. In 1902 he entered the I.M.S., studying at Netley under Sir Almroth Wright, obtaining a Scholarship in Surgery, and following this with the Gold Medal in Medicine. He accompanied Younghusband on his expedition to Thibet. For the details of his subsequent career we are indebted to the *Lancet*.

On return in 1905 he became assistant director of the Plague Research Laboratory, Bombay, and was associated with the work of the Indian Plague Commission. There he made what was probably his chief contribution to medicine in the discovery, in 1907, of the transmission of relapsing fever by the body-louse—a discovery that led Nicolle to demonstrate a similar method of conveyance in typhus. In 1908, at the request of the Government of India, he was appointed a member of the Royal Society's sleeping-sickness commission and worked for over two years in Uganda and Kenya on trypanosomiasis and its connection with the indigenous fauna. From now on his name appeared in the publications with that of his chief, Sir David Bruce, Lady Bruce and his friend and colleague of the R.A.M.C., Colonel A. E. Hamerton. In 1911 he was employed in special research on kala-azar in Assam.

Thereafter Mackie saw active military service in Baluchistan and during the 1914–18 war in France and Mesopotamia, being mentioned twice in dispatches and receiving the O.B.E. Returning to India in 1920 he became, successively, director of the Pasteur Institute, Shillong, and in 1923 of the Haffkine Institute, Bombay. In 1928 he was appointed chairman of the plague committee of the League of Nations and officiating public health commissioner to the Government of India, later officiating surgeon-general with the Bombay Government. Just before he retired in 1932 he acted once more as director of the Research Laboratory in Shillong. At this time he published, with B. P. B. Naidu, an important paper on the serum therapy of plague (*Lancet*, 1931, ii, 893). Mackie was representative of the Government of India at the Office International d'Hygiène Publique at Paris in 1919, 1922,

1926, and 1930 ; president of the tropical diseases section at the centenary meeting of the British Medical Association in July, 1925, and president of the medical and veterinary section of the Indian Science Congress in 1925. In 1933 he joined the staff of the Hospital for Tropical Diseases in London as pathologist and he remained there for five years. During that time he published papers on the pathology of the brain in trypanosomiasis (1935), and with Hamilton Fairley a contribution on a peculiar streptothrical infection hitherto undescribed. In 1937 he surprised his friends by taking, despite his age, the appointment of chief medical officer of the British Overseas Airways Corporation. In this capacity he travelled thousands of miles from one end of Africa to the other, not apparently in any way affected by heat or fatigue, and was able to his great delight to view from the air herds of game in those vast wild regions where, as a young man, he hunted them on foot. His main interest in recent years was in the prevention of yellow fever on aerodromes in Africa, and research into the disinsectization of aircraft.

Mackie was twice married and had three sons, one by his first wife and two by his second wife who survives him.

